# Multisensory effects of mask wearing on speech intelligibility and the benefit of multilingualism

**DOI:** 10.1590/2317-1782/20232022341en

**Published:** 2023-09-15

**Authors:** Filipa Ponte, Filipa Melo, Inês Duarte, Catarina Mendonça

**Affiliations:** 1 Departamento de Psicologia, Universidade dos Açores, Ponta Delgada, Portugal.; 2 Centro de Psicologia, Universidade do Porto, Porto, Portugal.

**Keywords:** Speech Perception, Stimuli, Vision, Audition, Pandemic, Covid, Perceção da Fala, Estímulos, Visão, Audição, Pandemia, Covid-19

## Abstract

**Purpose:**

Due to the pandemic of the Covid-19 disease, it became common to wear masks on some public spaces. By covering mouth and nose, visual-related speech cues are greatly reduced, while the auditory signal is both distorted and attenuated. The present study aimed to analyze the multisensory effects of mask wearing on speech intelligibility and the differences in these effects between participants who spoke 1, 2 and 3 languages.

**Methods:**

The study consisted of the presentation of sentences from the SPIN test to 40 participants. Participants were asked to report the perceived sentences. There were four conditions: auditory with mask; audiovisual with mask; auditory without mask; audiovisual without mask. Two sessions were conducted, one week apart, each with the same stimuli but with a different signal-to-noise ratio.

**Results:**

Results demonstrated that the use of the mask decreased speech intelligibility, both due to a decrease in the quality of auditory stimuli and due to the loss of visual information. Signal-to-noise ratio largely affects speech intelligibility and higher ratios are needed in mask-wearing conditions to obtain any degree of intelligibility. Those who speak more than one language are less affected by mask wearing, as are younger listeners.

**Conclusion:**

Wearing a facial mask reduces speech intelligibility, both due to visual and auditory factors. Older people and people who only speak one language are affected the most.

## RESUMO

**Objetivo:**

Devido à pandemia da doença Covid-19, o uso de máscaras em espaços públicos tornou-se comum. Ao cobrir a boca e o nariz, reduzem-se amplamente as pistas visuais associadas à fala, assim como se distorce e atenua o sinal auditivo. Este estudo teve como objetivo analisar os efeitos multissensoriais do uso da máscara na percepção da fala e a diferença entre participantes falantes de uma, duas ou três línguas.

**Método:**

Este estudo consistiu na apresentação de frases do SPIN teste a 40 participantes. Os participantes tinham como tarefa reportar as frases percebidas em quatro condições: Auditiva com máscara, audiovisual com máscara, auditiva sem máscara, audiovisual sem máscara. Conduziram-se duas sessões, com uma semana de intervalo, cada uma com os mesmos estímulos mas com diferente relação sinal-ruído.

**Resultados:**

Os resultados demonstraram que o uso de máscara reduz a inteligibilidade da fala, tanto devido à diminuição da qualidade do estímulo auditivo, como devido à perda de informação visual. A relação sinal-ruído afeta a inteligibilidade e com o uso de máscara são necessárias relações mais altas para obter qualquer identificação correta. Aqueles que falam mais do que uma língua, assim como os mais novos, são menos afetados na percepção de fala com uso de máscara.

**Conclusão:**

O uso de máscara facial reduz a inteligibilidade da fala, tanto devido a fatores visuais como auditivos. Indivíduos monolíngues, assim como os mais velhos, são os mais afetados nesta tarefa.

## INTRODUCTION

The emergence of the new variant of the SARS virus, the SARS-CoV-2, has drastically changed the way the population interacts socially in their daily lives due to the high level of contagion and increased mortality^(1)^. On March 11 2020 it was declared by the World Health Organization (WHO) that the virus had reached a sufficient level of spread and inaction to be declared a pandemic. Several restrictive measures have been taken to prevent its worsening, aiming to preserve public health and avoid population contamination. Of these measures, and with relevance for this study, was the compulsory use of the face mask whenever circulating in public places or in the presence of people. In the aftermath of the pandemic, the use of the face mask remains compulsory in some contexts.

Despite their benefits, the use of masks contributed to a new problem: the process around speech perception was altered. In cases of need for fast, effective, and understandable communication, such as in clinical contexts, there is now a deprivation of communication ability^(2)^. This phenomenon is due to the fact that, with a protecting layer covering the mouth, the propagation of sound is compromised, as it acts as an acoustic filter, which leads to speech signal distortion^(3)^. In addition, speech perception does not depend solely on the sound cues, but rather depends on audiovisual cue interactions^(4.)^. Visual cues are provided by the speaker through the articulatory movements they perform, while the auditory stimuli result from the propagation of the voice^(4)^. Several other factors interact with speech intelligibility, such as background noise. Background noise makes perception more difficult, in which case intelligibility will be facilitated by the ability to see the speaker^(5)^.

Multisensory interactions in speech perception may be demonstrated through some perceptual phenomena. When exposed to congruent visual articulation and auditory speech there is a speech reception facilitation effect, namely in noisy environments and when the subject has hearing loss (for a review, see Andersen et al.^(6)^). In a study by Skipper et al.^(7)^, neurophysiological activation and transcranial magnetic stimulation of the motor system during observation of mouth movements has been used to demonstrate the role of this system in multisensory speech perception.

With incongruent audiovisual stimuli, other effects arise, such as the McGurk Effect, where there is a categorical change in the perception of auditory speech^(8)^. The McGurk effect has been shown to be more pronounced in individuals who speak more than one language as opposed to in those who only speak one, which might be due to the fact that those who are bilingual have a greater knowledge and interaction with other phonemes than monolinguals. Furthermore, it is possible that the integration of audiovisual features is governed by different intramodal mechanisms in bilinguals^(9)^. In fact, bilingualism has been shown to have long-term consequences on some cognitive processes, namely increased cognitive control and improved metalinguistic awareness. However, studies have shown that bilinguals who use a second language generally have more difficulty understanding speech than monolinguals. This can be explained by the amount of time these subjects spend in both languages. According to Navarra and Soto-Faraco^(10)^, the speech comprehension of bilinguals is enhanced at the level of phonological processing through increased attention to visual stimuli.

Studies have also shown that bilingual individuals had an advantage over monolingual individuals in tasks with auditory language stimuli. This occurs in tasks in which interference needs to be suppressed in order to effectively process the target stimulus. Thus, bilinguals have an advantage in inhibitory control. Furthermore, the development of the auditory system may benefit when an individual is exposed to two different languages. According to the literature, it is common that there are no significant differences in the speech perception of bilingual persons compared to monolingual persons in a quiet environment. Some studies report that this occurs due to the determining parameter for speech recognition in silence, which is the threshold of audibility. In noisy situations, bilinguals show better results and this can be explained by the fact that the executive functions of inhibitory control, attention and memory are more evident in these individuals^(11)^.

In this study we were particularly interested in the interaction between visual and auditory cues in speech perception with and without mask, accounting for number of spoken languages and age bracket. In auditory-only speech perception with masks, adding a noisy background environment leads to what would be a mild high-frequency hearing loss, since masks stifle the higher frequencies of speech that help us differentiate similar sounds^(3)^. Regarding audiovisual speech perception with mask, a number of recent studies found that facial masks yield a negative effect on speech intelligibility^(5,12-16)^. All facial mask types hindered speech perception, mostly noisier environments, and with lower intelligibility scores in adults compared to young people^(5)^. These studies did not have an auditory-only condition, so direct comparisons between visual and audiovisual conditions could not be drawn. A study that did compare auditory-only speech perception and audiovisual speech perception with and without mask found that the 80% speech reception thresholds were increased by 2.5 dB when speech was presented with a simulated mask^(17)^. That study, however, only presented simulations of the masked speech, as opposed to actual talkers wearing a mask while speaking.

The purpose of the present study was to compare intelligibility scores with and without mask, in auditory-only and audiovisual conditions, in very noisy environments, and accounting for age group and number of languages spoken. The main research question was whether the mask affected speech intelligibility in auditory-only and in audiovisual conditions. It was hypothesized that it would be detrimental to both situations. It was also hypothesized that there would still be a benefit to the masked audiovisual condition, as opposed to the masked auditory-only condition, as some information is still conveyed by the uncovered half of the face. The second research question was whether participants of different ages and language abilities were affected differently in intelligibility scores of speech produced with the facial mask. It was hypothesized that younger people and people who speak more than one language might have higher intelligibility scores in the most difficult conditions.

## METHODS

### Participants

This study took place at the University of the Azores, in the Neurocognition Laboratory. Data were collected between September and November 2021. The research project was approved by the Ethical Committee of the University of the Azores and all participants provided written informed consent.

Initially, the sample consisted of 47 students attending the first year of the bachelor program in Psychology. The final sample consisted of 41 students, since 4 dropped out and the information of two of the subjects was declared as incomplete.

Prior to initiating the experiment, the participants filled out a questionnaire composed of the following topics: sociodemographic data, presence/absence of psychopathologies, frequency of interaction with individuals who use masks, languages spoken, frequency of computer use, presence/absence of visual or auditory deficits. The absence of hearing impairment was a participation requirement, and vision was required to be normal or corrected-to-normal. Among the participants, 87.8% were female, with only 12.2% being males. About 58% of the subjects were 25 years old or younger. In addition, 92.7% of the subjects had no psychopathologies and 7.3% had anxiety disorders. Most participants were used to listening to people wearing face masks (75%). 56% of the participants spoke only one language, 24% of the participants spoke 2 languages, and 15% of the participants spoke 3 languages.

### Stimuli

The stimuli consisted of videos of a subject speaking. Speech stimuli were 40 sentences taken from the *Spin* Test in European Portuguese^(18)^, which are all equivalent in length and intelligibility score. Videos were recorded using an iPad 2019, 7th generation 10.2 cm, which recorded both the image and sound stimuli. The videos were recorded in a controlled environment, acoustically treated and sound insulated. The environment was dark and a white diffuse light was used to ensure that all stimuli had the same brightness. Each video was edited to contain only the necessary length and had an average duration of 2 seconds each. Gaussian white noise was added to the background of all stimuli. In one session the background noise was louder, at 74 dB SPL, while in the other session the noise was 56 dB SPL. These levels are thought to simulate well two environments, one with a loud background, and the other a quieter indoor environment. Furthermore, in the unmasked condition, the speech signal was reproduced at 50 dB L_Aeq_ and with a mask it was at 46 dB L_Aeq_. This difference in level in the speech signal corresponded to the differences evaluated at the moment when the stimuli were recorded and is directly attributed to the effect of the mask on the sound. The type of mask used in all the videos was the surgical mask.

### Settings/Materials

The experiment was conducted in a room with sound insulation, with all walls and ceiling covered in dark acoustic foam, and with no lighting. A Windows computer was used to carry out the experiment, using the platform *Psychopy*. Auditory stimuli were presented through Marshall M-ACCS-00152 Monitor headphones. Visual stimuli were displayed on a 43-inch screen and participants' eyes were at 80 cm from the screen. The answers were collected by typing the perceived sentences on a dark keyboard.

### Procedures

There were four experimental conditions: Mask/NoVideo, Mask/Video, NoMask/NoVideo and NoMask/Video. In the Mask conditions (Mask/NoVideo and Mask/Video) the speaker was wearing a surgical mask, while in the NoMask (NoMask/NoVideo and NoMask/Video) conditions the speaker was unmasked. In the NoVideo conditions (Mask/NoVideo and NoMask/NoVideo), only the recorded sound was presented, while the screen remained dark.

In the Video conditions (Mask/Video and NoMask/Video), both the recorded sound and video were presented. The video was presented against a dark background. All 40 sentences of the SPIN test were rendered in all four conditions, totaling 160 stimuli. In order not to repeat sentences during the test, each participant was presented with only 10 sentences in each condition. Four groups of participants were created, each group with different sets of 10 sentences assigned to each of the four conditions. In other words, all subjects were exposed to the 40 sentences, but the condition of the sentences varied.

The experiment was assembled in *PsychoPy*, where each trial consisted of the random presentation of one sentence in one of the four conditions, and where, after each trial, participants wrote down the full sentence, as perceived.

As mentioned above, two sessions were administered, with a time interval of one week between each session to prevent habituation from occurring. Each session had different average durations, the high noise session lasted on average of 10 minutes, while the low noise session lasted on average of 20 minutes. This difference in duration was due mostly to the fact that participants were able to perceive more stimuli in the low noise session, and therefore spent more time typing the answers in that session.

### Data encoding

All responses were scored such that fully correct answers (the entire sentence was correct) corresponded 1 point, fully incorrect answers corresponded to 0 points, and partially correct answers corresponded to 0.5 points. This allowed for the obtention of a scale of proportion of correct answers ranging from 0 to 1.

## RESULTS

### General effects

**Figure 1:**
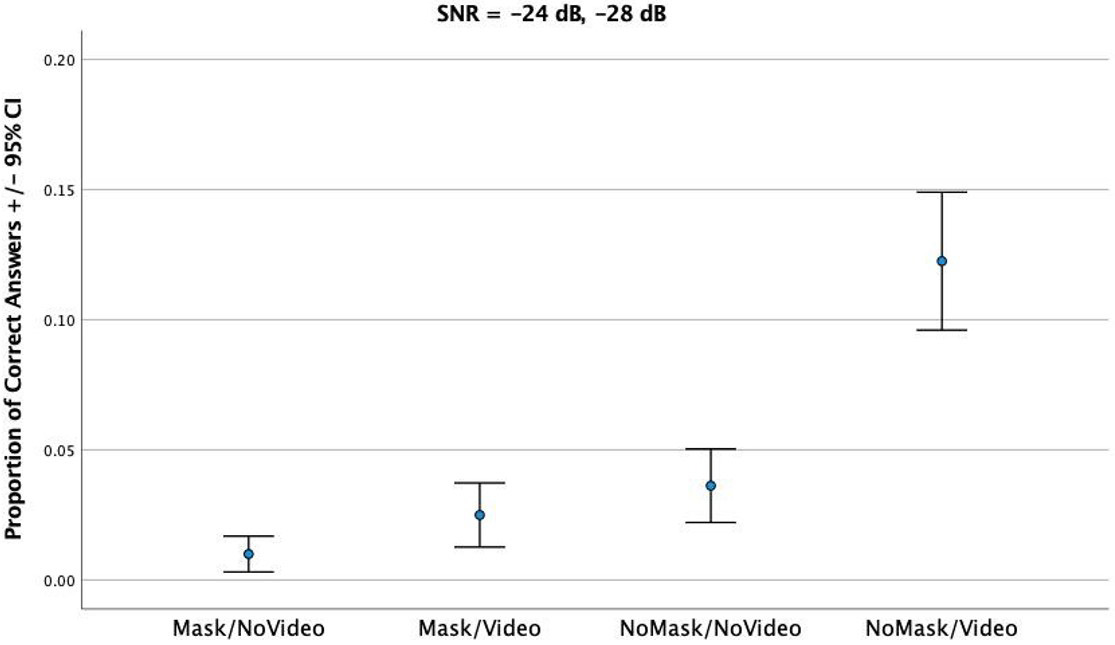
Proportion of Correct Answers for each condition in session 1

In the high noise session, with larger levels of background noise, speech recognition accuracy was overall poor. Figure 1 shows the results across the four experimental conditions. It can be observed that sentence recognition accuracy was lowest in the two conditions with mask, both without video (MEAN = 0.01, SD = 0.028) and with video (MEAN = 0.025, SD = 0.057). Without mask, accuracy was lower without video (MEAN = 0.036, SD = 0.071) and was highest with video (MEAN = 0.123, SD = 0.16).

In a Repeated Measures ANOVA, regarding data from Figure 1, accounting for all trials and experimental conditions as within-subject factors, a significant effect of condition was obtained (*F*_(3)_ = 8.800, *p* < 0.001). There was also a significant effect of repetition (*F*_(9)_ = 5.511, *p* = 0.019). An effect of participants was also observed (*F*_(39)_ = 6.186, *p <* 0.001), revealing significant differences between participants. Pairwise Tukey comparisons revealed that differences between conditions were only significant between the condition NoMask/Video and the remaining conditions. This reveals that both adding a mask and removing visual cues had a similar significantly negative impact on speech intelligibility scores in very noisy environments.

**Figure 2:**
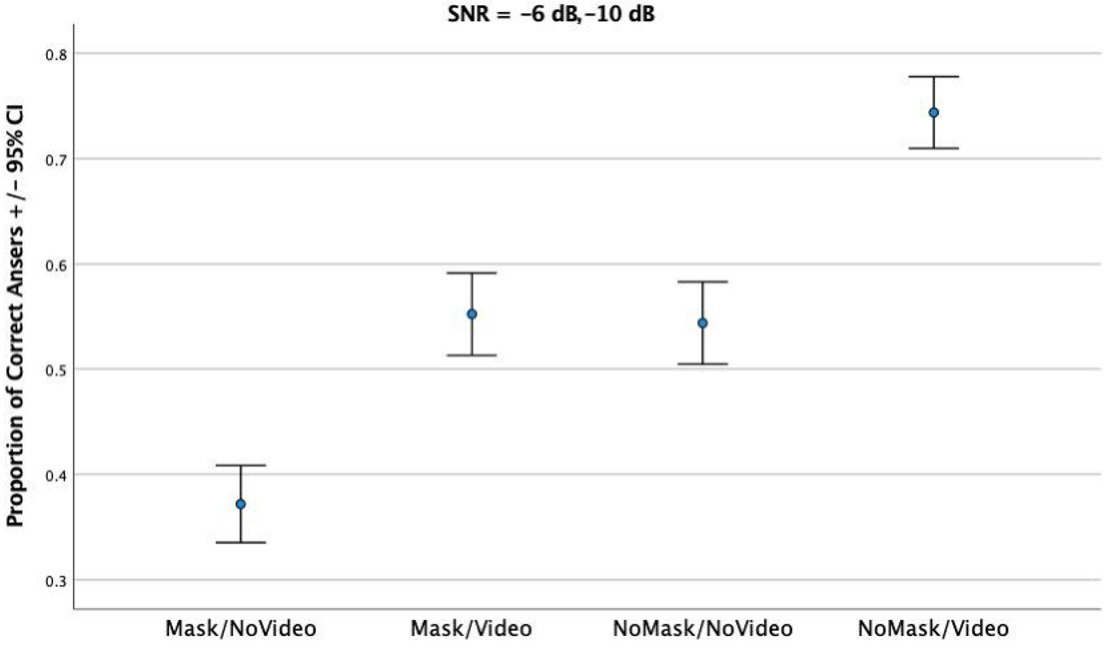
Proportion of Correct Answers for each condition in session 2

In the low noise session (Figure 2) accuracy rates in the speech recognition task were markedly higher. Again, the worst accuracy levels were obtained in the Mask/NoVideo condition (MEAN = 0.37, SD = 0.178), followed by both the Mask/Video (MEAN = 0.55, SD = 0.21) and NoMask/NoVideo (MEAN = 0.54, SD = 0.24) conditions. A clear superiority of the NoMask/Video was again observed in terms of accuracy (MEAN = 0.75, SD = 0.16).

To observe the effect of condition on the proportion of correct sentence recognition levels, regarding data from Figure 2, a Repeated Measures ANOVA was conducted, having experimental condition and repetition as within-subject effects. A significant effect of condition was obtained (*F*_(3)_ = 41.871, *p* < 0.001), and there was also a significant interaction between Condition and Repetition (*F*_(3)_ = 7.603, *p <* 0.001). Pairwise Tukey comparisons revealed that all experimental conditions differed significantly from each other, except for the pair Mask/Video and NoMask/NoVideo: Mask/NoVideo was significantly different from all other conditions; Mask/Video was significantly different from Mask/NoVideo and from NoMask/Video; NoMask/NoVideo was significantly different from Mask/NoVideo and NoMask/Video; and NoMask/Video was significantly different from Mask/NoVideo, and Mask/Video, NoMask/NoVideo. This seems to indicate that, in medium noise conditions, applying a mask has an effect as detrimental as removing the visual information, since the intelligibility scores in these two conditions were statistically dissimilar. There was a further summation effect, where combining mask and lack of visual stimulation further significantly lowered the intelligibility scores. Once again, a significant effect of participants was obtained, revealing significant differences between participants (*F*_(40)_ = 5.871, *p <* 0.001).

### Effects of age on speech perception

With the purpose of assessing whether participants with different ages had different results, the sample was split into two groups: age 25 and under, and over 25 years of age.

**Figure 3:**
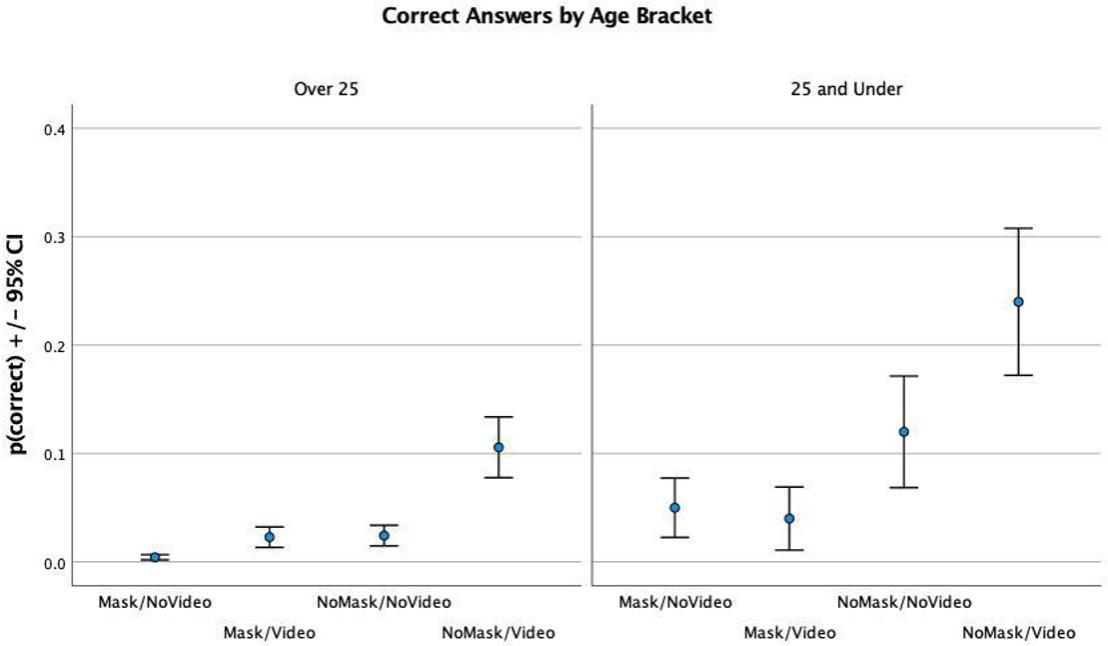
Correct Answers by Age Bracket

The average speech recognition scores in session 1 can be seen in Figure 3. It can be observed that younger participants had higher scores in all conditions.

A Repeated Measures ANOVA, regarding data from Figure 3, accounting for age bracket as between-subject effect, found a clear effect of age group in the first (noisy) block (*F*(1) = 9.107, *p* = 0.005). Post-hoc tests revealed that differences between age groups were significant in all experimental conditions. This effect of age was not observed in block 2 (*F*(2) = 0.193, *p* = 0.663). These results seem to indicate that age might be a mediating factor, but mostly in noisier conditions where speech intelligibility is a threshold, as seen in Figure 3.

### Effect of number of languages spoken

**Figure 4:**
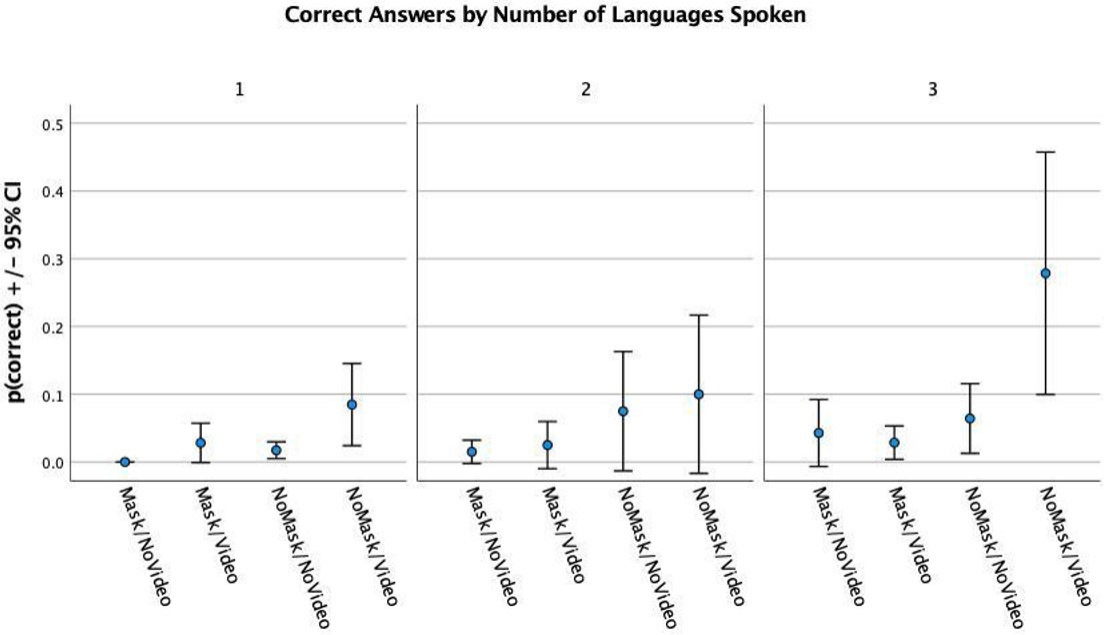
Correct Answers by Number of Language Spoken

To analyze the effect of the number of spoken languages on the speech recognition task, participants were reorganized into three groups, according to how many languages they spoke: 1 language, 2 languages, and 3 languages. Figure 4 shows the proportion of correct responses by number of languages spoken in the first session.

A benefit of polylingualism can be observed, where the higher the number of spoken languages, the higher the rates of correct speech recognition. While the benefit can be observed in all conditions, the largest differences are observed in the NoMask/Video condition. In a Repeated Measures ANOVA accounting for number of languages as between-subject factor and condition as within subject factor, a significant interaction between condition and number of languages was obtained (*F*(6) = 3.266, *p* = 0.006). A significant effect of number of spoken languages was also obtained (*F*(2) = 4.219, *p* = 0.023). Post-hoc Tuckey tests revealed that differences were only significant between one and three spoken languages.

In the second session, with less background noise, no significant effects were observed. In that session, there was no significant interaction between experimental condition and number of spoken languages (*F*(6) = 0.494, *p* = 0.812), and no significant effect of number of spoken languages (*F*(2) = 0.288, p = 0.751).

## DISCUSSION

In this study, it was verified that speech perception is altered when masks are used. There are additional relevant factors that interact with the mask wearing on speech intelligibility, namely noise level, age, and polylingualism.

In the session with high noise levels, participants obtained a higher proportion of correct answers in the condition without a mask and with video. Therefore, in very noisy conditions, both visual and auditory stimuli are necessary to achieve a degree of intelligibility. As hypothesized, in such noisy conditions, seeing the face, even if covered by a mask, was enough to provide significant speech intelligibility benefit. It is important to note that in this session the signal to noise ratio was very low (-24 dB), a level that had not been tested before in other studies. What distinguishes this session is that most participants operated at threshold, only being able to identify partial sentences, but rarely the full sentence.

Regarding the session with lower noise levels, once again, the mask-less condition with video yielded greater intelligibility. The Mask/NoVideo condition obtained a significantly lower percentage of correct sentence identification, and we can conclude that applying a mask has as detrimental an effect as removing visual information. A significant effect of participants was obtained, revealing interindividual differences. The fact that background noise interacts with mask-wearing in its effects on speech perception has been demonstrated before^(13,16)^ and was replicated here.

Regarding the effect of age, there was also a significant effect on speech intelligibility in the noisier session. This finding was also demonstrated in the study by Brown et al.^(5)^, where a model that included age, mask type and noise level indicated that adults had lower speech intelligibility as compared to young people. One possible mechanism to explain the effect of age observed here might be age-related hearing loss, which can lead to lower speech intelligibility scores in general, as well as an increase in the hearing thresholds^(19)^. Indeed, in both the unmasked conditions (with and without video) we could observe marked differences between both age groups. Another hypothesis would be that older individuals take longer to learn and adapt to changes due to reduced neuroplasticity^(20)^. Older subjects would therefore take longer to learn to extract speech cues from mask-wearing speakers. There were small, but significant, differences between the age groups in the masked conditions, which could support this assumption to an extent. Further studies should better control for the amount of experience with masked stimuli to detangle these effects.

Finally, there was an effect of multilingualism on speech intelligibility in the noisiest session, and it can be concluded that subjects who speak more than one language decode the speech signals better. This is in accordance with previous literature demonstrating that bilingual individuals have more developed executive functions of inhibitory control, attention, and memory, compared to monolingual individuals^(11)^. These subjects have greater sensitivity to audiovisual speech stimuli and greater accuracy in phoneme perception, which consequently leads to a greater activation of speech-related neuronal networks^(10-12)^. It is therefore hypothesized that the benefit observed here was related to improved executive function in general, and phoneme processing ability in particular, in those who speak more than one language.

Our experiment is conceptually similar to other recent experiments that found relative decreases in speech intelligibility with the use of face masks^(5,12-17)^. However, this study is the first to compare directly auditory-only with audiovisual conditions regarding the effects of mask wearing on speech intelligibility with real stimuli. The removal of visual stimuli has an effect similar to the application of the mask on speech intelligibility, but both factors combined have cumulative detrimental effects. The visual stimulus, even when wearing a mask, still conveys relevant information that is able to improve the intelligibility of spoken sentences.

There were some limitations in our study. It would be important to have a greater number of participants, with greater representation of older age groups. The effect of mask wearing on speech intelligibility should be assessed carefully in participants with hearing loss and in older participants, as it is reasonable to assume these would be the populations experiencing greater difficulties.

Public health policies regarding mask wearing should be informed by, and account for, the trade-offs of mask wearing and communication impairment in different contexts and with different populations.

## CONCLUSION

The present study reveals that mask wearing affects speech intelligibility in noisy contexts both with and without the presence of visual information. It is not enough to see the speaker, the mask must be off to achieve maximum intelligibility. Even when there is no image, such as when speaking on the phone, taking off the mask is beneficial for speech intelligibility. Older adults and people who speak only one language are the most affected by mask wearing in their ability to understand speech.
